# DSB profiles in human spermatozoa highlight the role of TMEJ in the male germline

**DOI:** 10.3389/fgene.2024.1423674

**Published:** 2024-07-08

**Authors:** Maurice Scheuren, Jonas Möhner, Max Müller, Hans Zischler

**Affiliations:** ^1^ Division of Anthropology, Faculty of Biology, Institute of Organismic and Molecular Evolution, Johannes Gutenberg University Mainz, Mainz, Germany; ^2^ Institute for Pharmaceutical and Biomedical Sciences, Johannes Gutenberg-University Mainz, Mainz, Germany

**Keywords:** DNA repair, chromatin, epigenetics, human sperm, double-strand breaks, TMEJ

## Abstract

The male mammalian germline is characterized by substantial chromatin remodeling associated with the transition from histones to protamines during spermatogenesis, followed by the reversal to nucleohistones in the male pronucleus preceding the zygotic genome activation. Both transitions are associated with the extensive formation of DNA double-strand breaks (DSBs), requiring an estimated 5 to 10 million transient DSBs per spermatozoa. Additionally, the high transcription rate in early stages of spermatogenesis leads to transcription-coupled damage preceding meiotic homologous recombination, potentially further contributing to the DSB landscape in mature spermatozoa. Once meiosis is completed, spermatozoa remain haploid and therefore cannot rely on error-free homologous recombination, but instead depend on error-prone classical non-homologous end joining (cNHEJ). This DNA damage/repair-scenario is proposed to be one of the main causes of the observed paternal mutation propensity in human evolution. Recent studies have shown that DSBs in the male pronucleus are repaired by maternally provided Polθ in *Caenorhabditis elegans* through Polθ-mediated end joining (TMEJ). Additionally, population genetic datasets have revealed a preponderance of TMEJ signatures associated with human variation. Since these signatures are the result of the combined effect of TMEJ and DSB formation in spermatozoa and male pronuclei, we used a BLISS-based protocol to analyze recurrent DSBs in mature human sperm heads as a proxy of the male pronucleus before zygotic chromatin remodeling. The DSBs were found to be enriched in (YR)_n_ short tandem repeats and in evolutionarily young SINEs, reminiscent to patterns observed in murine spermatids, indicating evolutionary hotspots of recurrent DSB formation in mammalian spermatozoa. Additionally, we detected a similar DSB pattern in diploid human IMR90 cells when cNHEJ was selectively inhibited, indicating the significant impact of absent cNHEJ on the sperm DSB landscape. Strikingly, regions associated with most retained histones, and therefore less condensed chromatin, were not strongly enriched with recurrent DSBs. In contrast, the fraction of retained H3K27me3 in the mature spermatozoa displayed a strong association with recurrent DSBs. DSBs in H3K27me3 are associated with a preference for TMEJ over cNHEJ during repair. We hypothesize that the retained H3K27me3 may trigger transgenerational DNA repair by priming maternal Polθ to these regions.

## 1 Introduction

A central tenet of human evolutionary biology is the paternal bias in germline mutations, which is frequently observed in various mammalian taxa. Historically, the textbook explanation for the paternal mutation propensity is that it results from the greater number of cell divisions - and thus DNA replication cycles—that are required to complete spermiogenesis as compared to oogenesis. However, a number of recent findings have questioned this view, emphasizing the role of DNA damage and DNA repair in spermatozoa and zygotes in generating *de novo* mutations (Link et al., 2017). Canonical cell functions and environmental influences pose a threat to the integrity of a cell’s genetic information with DNA double-strand breaks (DSBs) representing the most lethal form of damage. In context of gametogenesis, the mammalian male germline stands out transcribing almost the entire genome during early stages of spermatogenesis, enabling a “*transcriptional scanning*,” but may also create ample opportunities for transcription-coupled damage (Dudás and Chovanec, 2004; Soumillon et al., 2013; Xia et al., 2020). Proceeding further into meiosis, intentional DSBs are generated as a prerequisite for exchanging genetic material in the prophase of meiosis. In addition, postmeiotic DSBs have been detected during spermatogenesis facilitating the histone-to-protamine transition, in which histones are largely but not completely replaced by the more basic protamines PRM1 and PRM2. It is hypothesized that during chromatin remodeling, free DNA supercoils are formed during histone retrieval. This process also requires the generation of transient DSBs to accommodate the necessary topological restructuring. The exact nature of these DNA breaks, whether single-strand or double-strand breaks, is still under scrutiny (Marcon and Boissonneault, 2004; Hammoud et al., 2009; Bao and Bedford, 2016).

In general, DSBs are repaired through either homologous recombination (HR), as seen, e.g., during meiosis, or non-homologous end joining (NHEJ). The latter can be further divided into diverse pathways that differ in the composition of the individual factors and are dubbed classical NHEJ and alternative EJ (a-EJ). In contrast to HR, the end joining pathways are error-prone due to end resection and subsequent indels or reliance on microhomologies in the range of 2–6 bp only, possibly giving rise to erroneous rejoining of distant DSBs. In mammals, the prominent a-EJ is dependent on DNA polymerase θ (Polθ), a unique DNA-polymerase-helicase fusion protein with an inactive proofreading domain and the very remarkable feature of additionally possessing RNA-dependent DNA polymerase activity *in vitro* (González-Marín et al., 2012; Wyatt et al., 2016; Chandramouly et al., 2021).

Both, cell type and developmental stage influence the balance between specific DSB repair pathways. Thus, differentiated somatic cells often resolve DSBs through NHEJ, while embryonic stem cells preferentially use HR. As round spermatids are haploid cells, DSBs cannot be repaired through homology-based mechanisms. Instead, DSBs must be repaired by error-prone mechanisms such as non-homologous end joining (NHEJ) or microhomology-mediated end joining (MMEJ), most likely involving Polθ (Theta-mediated end joining, TMEJ) (Derijck et al., 2008; Mathiasen and Lisby, 2014; Wyatt et al., 2016; Chang et al., 2017). Paternal DSB/repair profiles and thus the *de novo* mutations from a paternal perspective, are also influenced by DNA breaks generated during zygotic reprogramming, specifically during the protamine-to-histone transition. γ-H2AX foci, a surrogate marker for DSBs, are detected at the same time of paternal DNA demethylation suggesting that they most likely arise in the separate male pronucleus and during zygotic reprogramming (Wossidlo et al., 2010). Indeed, Wang and colleagues (2023) demonstrated in *Caenorhabditis elegans* that paternal DNA damage can be repaired in the zygote by maternally provided Polθ. Upon analyzing data from the 1,000 Genomes Project and the Polaris dataset, they moreover found a preponderance of the TMEJ signatures in the *de novo* mutation profiles suggesting that human variation is significantly determined by the paternal DSB landscape and its repair. Obviously, this profile is the net outcome of the mature spermatozoa DSB landscape and the DSBs induced in early zygotic reprogramming, along with its pre- and postzygotic repair in the mature sperm and the paternal pronucleus (Dolzhenko et al., 2017; Byrska-Bishop et al., 2022; Wang et al., 2023).

In this study, we used mature human sperm heads as a proxy to the male pronucleus before zygotic reprogramming and thus before reconstituting the nucleo-histone profiles. This approach allowed us to disentangle the relative contribution of DSBs and their repair during spermatogenesis separating it from DSB induction/repair during early zygotic reprogramming. Therefore, we applied a modified BLISS-based protocol on mature human sperm heads that were isolated by differential lysis of human ejaculate samples. The results highlight an association between recurrent DSBs and (YR)_n_ short tandem repeats and a colocalization with evolutionarily young SINEs, similar to previous observations in murine spermatids (Burden et al., 2023). Additionally, the recurrent DSBs displayed a large positive association with retained H3K27me3 in the mature spermatozoa, which facilitates repair through TMEJ rather than NHEJ, implicating a transgenerational mechanism from “*poised*” DSBs in mature spermatozoa to TMEJ-repaired DSBs in the male pronucleus. By selectively inhibiting classical NHEJ in diploid human IMR 90 cells, we observed a DSB pattern similar to that found in haploid spermatozoa, thus supporting our findings of characteristic spermatozoa DSB patterns in the absence of cNHEJ.

## 2 Material and methods

### 2.1 Cell culture of IMR90 cells and inhibition

IMR90 cells were cultured in Dulbecco’s modified Eagle’s medium supplemented with 10% fetal bovine serum (Thermo Fisher Scientific) at 37°C and 5% CO_2_. To inhibit DNA-PKc activity in the IMR90 cells, AZD7648 (MedChemExpress) was dissolved in DMSO and added to the medium at a final concentration of 1 μM, while an equivalent volume of DMSO was added to the control medium. After 48 h of incubation, the cells were harvested for further processing.

### 2.2 Isolation of mature sperm heads

The ejaculate samples were obtained non-invasively from volunteers who provided them through masturbation after 2–3 days of sexual abstinence, with informed consent. All samples were liquefied for at least an hour and further processed.

The total ejaculate was centrifuged (16,000 × *g*, 5 min at room temperature) and the supernatant was discarded. The cell pellet was resuspended in 1.35 mL of lysis buffer (10 mM TRIS pH 8; 10 mM EDTA; 100 mM NaCl; 4% SDS). The suspension was centrifuged, the supernatant discarded. The procedure was repeated once more, and the resulting pellet was utilized for downstream protocols.

### 2.3 DNA double-strand breaks detection

Two distinct cell types were used for the breakome analysis, namely isolated sperm heads and cultured IMR90 cells. The BLISS experiments were performed as described elsewhere, with slight modifications adjusted to the characteristics of mature spermatozoa (Yan et al., 2017; Bouwman et al., 2020). Briefly, isolated sperm heads from a total ejaculate or roughly 2 × 10^6^ cultured IMR90 cells were resuspended in 1X PBS and mixed with an equal volume of 1.5% low melting point agarose before being drawn up with a syringe. To start, approximately 20 µL of gel was submerged in buffer (75 mM NaCl, 25 mM EDTA; pH 8) supplemented with dithiothreitol to achieve a final concentration of 1 mM and incubated for 1 h at room temperature. Next, the buffer was exchanged with lysis buffer (25 mM EDTA, 1% SDS; pH 8) supplemented with 1 µL of Proteinase K and incubated overnight (15–19 h) at room temperature. The following day, the genomic DNA was blunted using the Quick Blunting Kit (New England Biolabs). Agarose was digested using β-Agarase I (New England Biolabs) prior to Adapter ligation. Next, the adapter containing the RNA polymerase promoter was ligated with T4 DNA ligase (New England Biolabs) and incubated overnight (15–19 h) at 16°C. Unligated adapters were removed using gel electrophoresis and the gDNA was recovered using the QIAquick Gel Extraction Kit (Qiagen). The isolated gDNA was fragmented using Covaris S-series with “Duty Factor 10%, Intensity 4 and Burst 200” for 80 s. The *in vitro* transcription reaction was carried out at 37°C overnight (15–19 h) using the HiScribe T7 Quick High Yield RNA Synthesis Kit (New England Biolabs). The resulting RNA was polyadenylated with *Escherichia coli* Poly(A) Polymerase (New England Biolabs), reverse transcribed using the Biozym cDNA Synthesis Kit (Biozym), and then amplified using PCR with the Taq PCR Core Kit (Qiagen). The NEBNext Ultra II DNA PCR-free Library Prep Kit for Illumina (New England Biolabs) was used by Novogene to prepare and sequence the PCR-free libraries employing a PE150 strategy on a NovaSeq 6000 (Spermatozoa *n* = 2, AZD7648 treated IMR90 cells *n* = 2, untreated IMR90 cells *n* = 2).

The generated NGS reads were scanned for the first and second barcode sequences, allowing for one mismatch, subsequently the enclosed UMI and adjacent sequence were extracted. The resulting sequences were mapped to the GRCh38 reference genome using Bowtie2 in PE mode with options “—local -N 1 –fr” and then converted to bed-format using SAMtools. To obtain unique DSB events, PCR duplicates were singularized by identifying alignments that share a starting position within a ±6 bp of range and a Hamming distance < 1 regarding the UMI. The resulting DSB positions were depleted from blacklisted regions using BEDTools intersect (Quinlan and Hall, 2010) and the reference “GRCh38-blacklist.v2.bed” (https://github.com/igordot/reference-genomes/blob/master/GRCh38/blacklist.v2.bed). To subsequently identify genomic regions enriched in DSBs, a topological-based workflow was developed as a model-free method, following the proposal by Mitra et al. (2020). Therefore, we identified local maxima in the genome wide DSB distribution. In summary, BEDTools coverage with the option “-counts” was used to calculate DSB coverage over 100 bp sliding 1 kb bins of GRCh38. The resulting smoothed DSB distribution was used to identify local maxima within the distribution using “scipy.signal.find_peaks” from the SciPy package (Virtanen et al., 2020). The replicates’ resulting local maxima were used to identify common maxima through BEDTools intersect. These common local maxima were centered into a 1 kb overlapping shared “peak” and were subsequently referred to as recurrent DSB clusters (RDCs). HOMER’s “annotatePeaks.pl” was used to analyze the resulting RDCs for annotation, with the hg38 reference (Heinz et al., 2010). The RDCs were correlated with Z-DNA, short tandem repeats, and G-quadruplexes (https://nonb-abcc.ncifcrf.gov/apps/Query-GFF/feature/) (Cer et al., 2012). MISA was used to analyze short tandem repeats, only allowing 2 bp repeat units and a maximum gap of 3 bp (Thiel et al., 2003; Beier et al., 2017). The PRDM9 binding sites were identified using HOMER’s “findMotifsGenome.pl” and the degenerated PRDM9 binding motif “ccn ccn tnn ccn c,” in addition to active recombination hotspots being analyzed (Pratto et al., 2014). The associations between the RDCs and chromatin states were analyzed using BEDTools multiinter. The resulting sets, which included the RDCs, were visualized using the R packages “ComplexHeatmap” and “UpSetR” (Gu et al., 2016; Conway et al., 2017). The significance of all associations was calculated using the permutation test (*n* = 1,000) of the R package “regioneR” with unmasked GRCh38 for randomization (Gel et al., 2016).

### 2.4 Publicly available data

The work employed data from the Gene Expression Omnibus, which was converted to GRCh38 using UCSC Liftover if necessary. The dataset PRJNA480448 was obtained from the Sequence Read Archive and reanalyzed according to ENCODE standards for the corresponding sequencing strategy (Supplementary Table S4) (Hammoud et al., 2014; Lesch et al., 2016; Jung et al., 2019; Bae and Lesch, 2020; Oikawa et al., 2020; Scheuren et al., 2023).

## 3 Results

### 3.1 Genomic distribution of RDCs in mature spermatozoa

Mature spermatozoa are a unique cell population for analyzing DSBs because they are mainly transcriptionally dormant and only contain a haploid genome, which is assumed to be highly compacted to reach the oocyte intact. However, *in vivo* the DNA of mature spermatozoa is generally fragmented to some extent (Aitken and Iuliis, 2010). Therefore, the above-mentioned sperm characteristics may give rise to unique patterns of DNA damage that contribute to the DSB landscape in comparison to somatic cells. To identify common hotspots of DNA damage across individual variability, which is influenced by many external and internal factors, we used NGS-DSB data to identify recurrent DSB clusters. We used mature human sperm heads isolated from total ejaculate to label the present DSBs *in situ* and subsequently sequenced them using a modified BLISS method to account for the characteristics of this particular cell type. The NGS data was scanned for adapter sequences and processed into unique DSBs for subsequent analysis. To identify clusters of recurrent DSB formation in a model-free method as proposed by Mitra et al. (2020), we established a topological method by identifying regions corresponding to local maxima of DSBs in the genome-wide DSB distribution that exhibited significantly elevated levels relative to the surrounding distribution.

**FIGURE 1:**
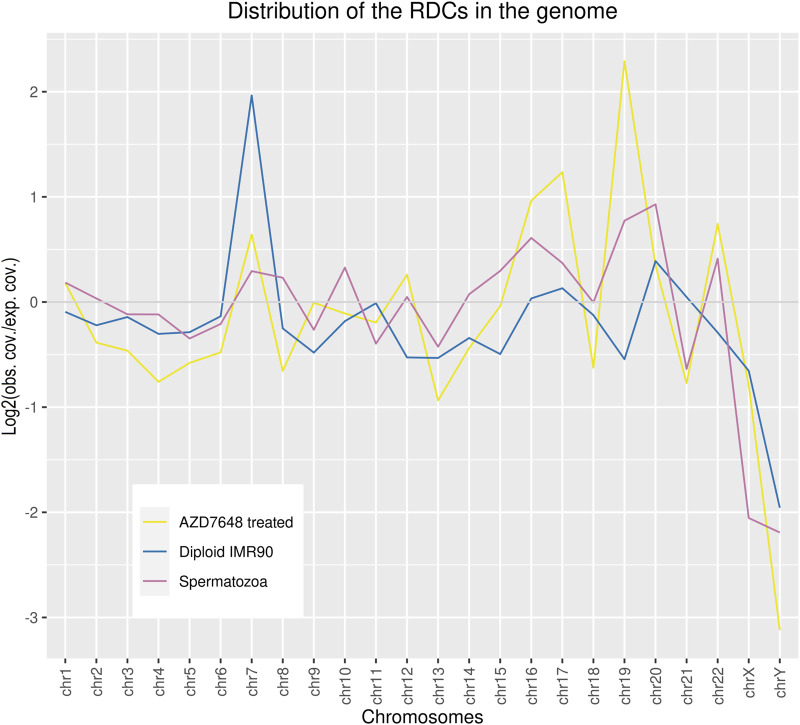
The relative coverage of RDCs across the chromosomes was calculated by setting the RDC coverage per chromosome (observed) in proportion to the corresponding chromosomes’ size in the genome (expected). This resulted in log2 (observed coverage/expected coverage) values of the RDC coverage in the genome.

**FIGURE 2:**
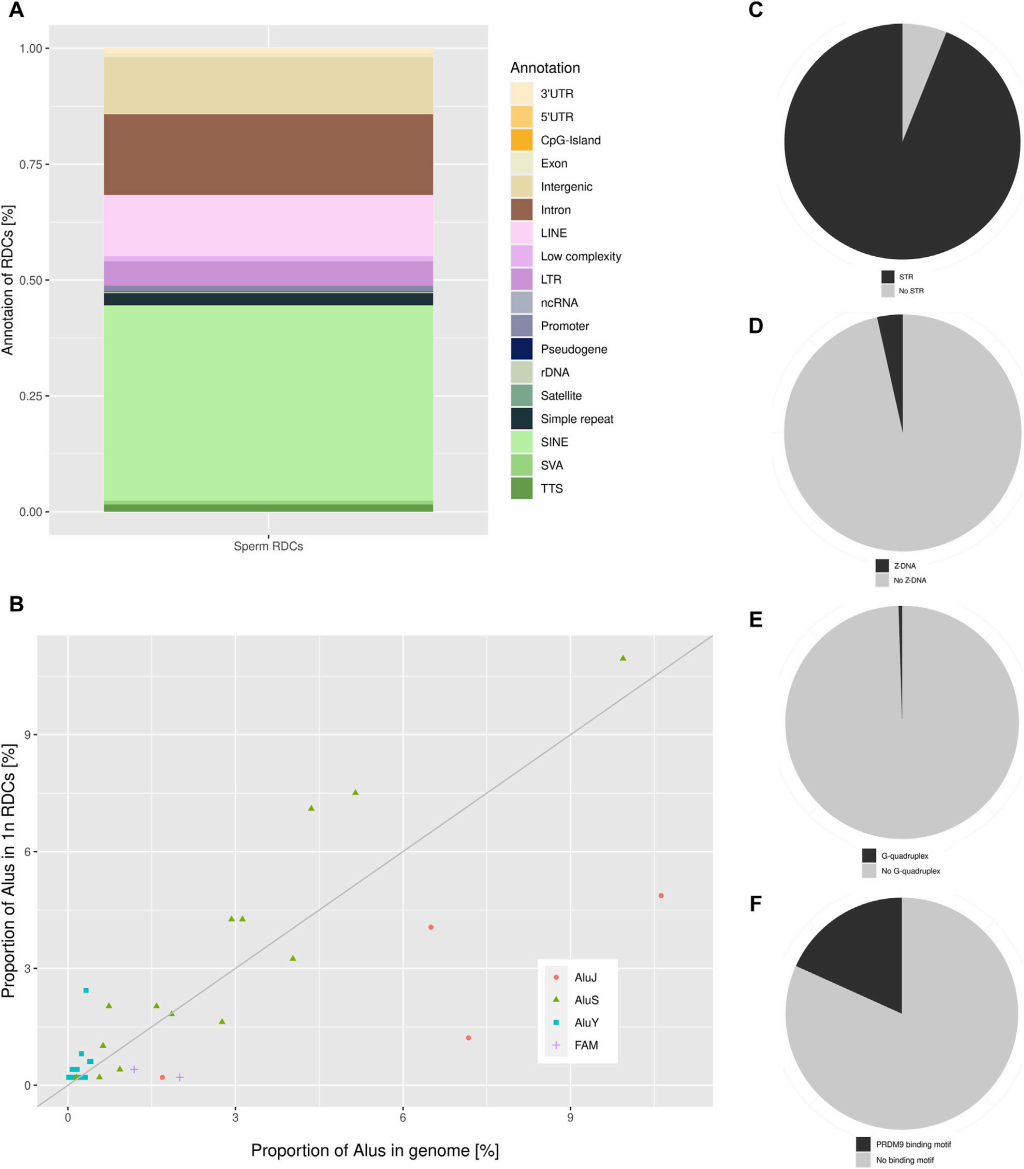
**(A)** The detailed annotation of the RDCs in sperm as generated by Homer, depicted as a percentage of total RDCs (% of total RDCs). **(B)** The distribution of the sperm RDCs across various Alu subfamilies within the human genome is depicted as a proportion of Alu subfamilies located in the RDCs on the *y*-axis, and the relative proportion of the corresponding Alu-subfamily of all genomic Alu elements in the genome on the *x*-axis. The bisection indicates an equal distribution of expected and observed Alu-subfamilies in the RDCs corresponding to their genomic representation. Pie charts displaying the proportion of sperm RDCs overlapping **(C)** STRs, **(D)** Z-DNA, **(E)** G-quadruplexes, and **(F)** PRDM9 binding sites.

A total of 1,233 RDCs were detected in the mature spermatozoa, with an average GC content of 41.29%, consistent with the genome’s average GC content (Piovesan et al., 2019). The distribution of RDCs across the chromosomes was calculated by setting the RDC coverage per chromosome in proportion to the corresponding chromosome size, resulting in log2 (observed/expected) values of the RDC distribution in the genome. The majority of RDCs are evenly distributed across the genome as expected, although some outliers were identified. The RDC display an underrepresentation on both sex chromosomes and chr21, and an overrepresentation on chr16, chr19, chr20, and chr22 (Figure 1). To investigate the origins of the RDCs further, we used the Homer tool to annotate the detected RDCs. The top-level annotation shows that most RDCs are located in introns (47.93%) and intergenic regions (47.28%). As introns and intergenic regions exhibit various compositions of repetitive elements, particularly transposable elements (TE), we further investigate the detailed annotation generated by Homer. The detailed annotation reveals that many RDCs are indeed located in true intronic sequences and intergenic regions (17.11% and 12.08%, respectively). However, more than half of the RDCs are found in short interspersed nuclear elements (SINEs) and long interspersed nuclear elements (LINEs) located in these regions (41.44% and 13.06%, respectively). It is noteworthy that over 40% of RDCs present in mature spermatozoa are located in SINEs, specifically Alu elements (Alus) (Figure 2A). Since the major chromatin changes during spermatogenesis enable a global transcription of the sperm genome, including TEs, with the potential activation of various Alus and the fact that Alus are abundant in the human genome and display varying frequencies of activity throughout primate evolution, our investigation focused on the relationship between Alus and RDCs (Batzer et al., 1996; Davis et al., 2017). The Alu phylogeny comprises of three major subfamily branches, with AluJ being the oldest, followed by AluS, and the youngest being AluY, each differentiated into various sub-branches. To investigate the distribution of RDCs in the Alu phylogeny, we used RepeatMasker (Smit et al., 2008) annotations to calculate the proportions of all Alu subfamilies within the genome and compared them to the corresponding observed distribution in the RDCs. It is noteworthy that almost all observed AluY elements are overrepresented in the RDCs compared to the genomic frequency, while over two-thirds (68.75%) of AluS elements show overrepresentation. This suggests a negative correlation between DSB formation and the age of the corresponding Alu subfamily, which also correlates with the activity of Alu subfamilies. The subfamilies with the highest overrepresentation are AluYh7, AluYa5, AluYb9, AluYk2, and AluYb8. In contrast, the oldest major subfamily branch, AluJ, and ancestral Alus are mostly depleted in the RDCs. AluSc5, FLAM_A, AluJr, AluJr4, and FLAM_C display the largest underrepresentation (Figure 2B).

Since DSB-data of murine spermatids suggests a strong association between DSBs and short tandem repeats (STRs), we further investigated this association in the DSB landscape of mature human sperm (Burden et al., 2023). Therefore, we used annotated STRs in the human genome to identify significant associations with the RDCs using permutation testing. Strikingly, the RDCs and STRs display a strong positive association, with 93.92% of them containing at least one STR (*p*-value < 0.05, *Z*-score: 35.94) (Figure 2C). To investigate the STR motifs, MISA was used with the analysis restricted to repetitive units of 2 bp in length and a maximal gap of 3 bp between two units. The results show a preference for alternating purine and pyrimidine bases with (YR)_n_ in 37.82% and (RY)_n_ in 27.51% of STRs. The single most common repetitive unit is (CA)_n_, which accounts for 21.69% of all associated STRs, also displaying the strongest association with the DSBs observed in the murine spermatozoa. In general, STR motifs, particularly those with alternating purine and pyrimidine bases, can lead to the formation of non-B DNA structures, including Z-DNA, which is potentially less stable than canonical B-DNA. Therefore, we analyzed the association of the corresponding RDCs with predicted non-B DNA structures. However, only a few RDCs are located in regions of potential Z-DNA (3.49%) and no significant association can be observed (*p*-value > 0.05, *Z*-score: 0.35) (Figure 2D). Regarding G-quadruplexes, a common Non-B DNA structure predominantly found in promoters and telomeres, the RDCs display little overlap (0.49%) and as a result, there is no significant association observed with predicted G-quadruplex (*p*-value > 0.05, *Z*-score: −0.94) (Figure 2E).

A common spermatogenesis-specific motif in context of intentional DSB induction is the binding motif of PRDM9. These motifs are located in designated “*hotspots*” for meiotic recombination, where PRDM9 guides DSB induction of the cofactor SPO11 during meiosis (Grey et al., 2018). Therefore, we investigated the association between the RDCs and putative PRDM9 binding sites. The DSBs are induced by SPO11 in approximately 150 bp flanking the motif, and the predicted genomic binding sites were correspondingly extended on both sides. We observed a positive correlation between the PRDM9 binding sites and the RDCs (*p*-value < 0.05, *Z*-score: 7.35), with approximately 18.25% of the RDCs displaying a PRDM9 motif in close proximity (Figure 2F). As most active recombinational hotspots are generally located in “*gene deserts*,” we annotated the PRDM9 associated RDCs regions and 46.67% of these are located in intergenic regions. Since not all PRDM9 binding events result in the induction of DSBs, we analyzed the association of the RDCs with active recombination hotspots for individuals with the most common PRDM9 allele identified by Pratto and colleagues (2014). However, only a small proportion of RDCs overlap with these recombination hotspots, and no significant association can be observed (1.95%, *p*-value > 0.05, *Z*-score: 0.39).

### 3.2 Sperm RDCs and chromatin

The structure of chromatin affects both the accessibility and stability of DNA. Therefore, we investigated the chromatin states of the RDCs. The histone-to-protamine transition is tightly regulated by different stages during spermatogenesis distinguished by specific histone variants and their PTMs and is in general considered to be incomplete. The resulting retained histones in the sperm genome are extensively investigated in the context of “*epigenetic inheritance*” and fertility (Hammoud et al., 2009; Torres-Flores and Hernández-Hernández, 2020). To obtain a comprehensive overview of the interactions between RDCs and chromatin states, publicly available data were used to compute a combination matrix via the R package “*ComplexHeatmap*.” The largest and most significant sets containing RDCs were further investigated.

**FIGURE 3:**
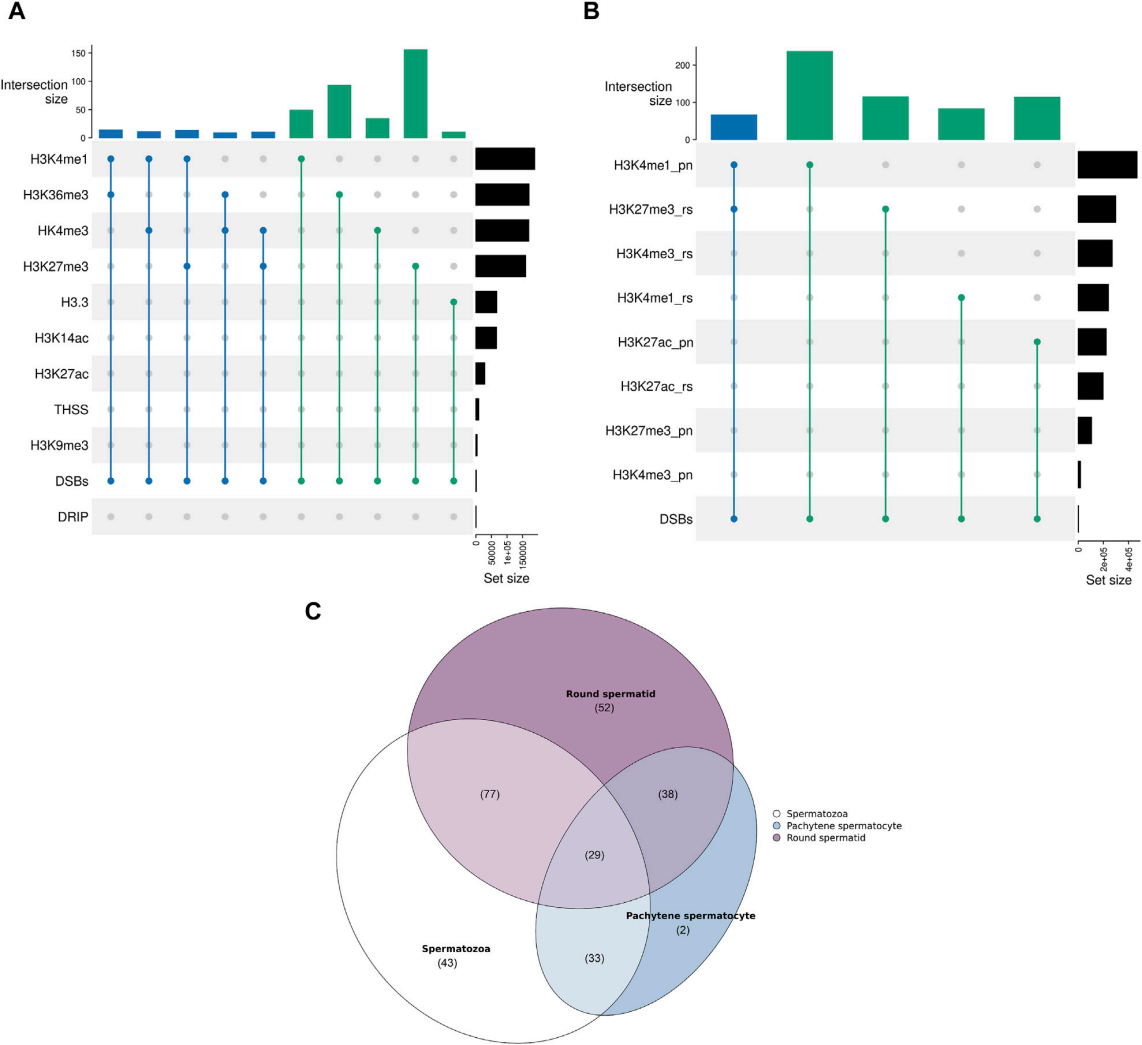
**(A)** Upset plot displaying the shared recombinational overlaps between the sperm RDCs and various chromatin states found in mature spermatozoa. The upper plot shows the absolute number of overlaps, while the right plot displays the size of the corresponding chromatin state [kb] as a reference. The degree of the overlap is color-coded (*n* = 2 in green and *n* = 3 in blue). “DRIP” indicates the R loop data obtained through DNA-RNA immunoprecipitation. **(B)** Upset plot displaying the shared recombinational overlaps between the sperm RDCs and various chromatin states during spermatogenesis. The upper plot shows the absolute number of overlaps, while the right plot displays the size of the corresponding chromatin state [kb] as a reference. The degree of the overlap is color-coded (*n* = 2 in green and *n* = 3 in blue). “pn” indicates pachytene stage and “rs” indicates roundspermatid stage. **(C)** Venn diagram presenting the degree of overlap between the RDCs and H3K27me3-associated regions during the round spermatid stage, pachytene stage, and in mature spermatozoa. The parentheses indicate the size of the overlap.

The RDCs show the largest intersection with H3K27me3, also displaying a positive association (*p*-value < 0.05, *Z*-score: 8.43). The corresponding regions are mainly intergenic (76.28%), with 21.79% of these sets located in Alus and 12.82% in LINEs. The second-largest overlap and positive association is observed with H3K36me3 (*p*-value < 0.05, *Z*-score: 3.33) and primarily occurs within intronic sequences (87.1%). Interestingly, H3K36me3 can potentially be methylated by PRDM9 during spermatogenesis and 10.75% of the corresponding sets contains a PRDM9 binding motif in near proximity, suggesting a possible association. Additionally, there is a larger overlap and positive association with H3K4me1 (*p*-value < 0.05, *Z*-score: 2.74). The detailed annotation also indicates that the corresponding RDCs are mostly located in Alus (36.0%) and introns (25.33%). The relationship between the RDCs and spermatogenesis relevant H3.3 is the only significant negative association (*p*-value < 0.05, *Z*-score: −1.67). Interestingly, there is no significant overlap or positive correlation with Tn5 transposase hypersensitive sites (THSSs) (*p*-value > 0.05, *Z*-score: −0.81) or R-loop data (*p*-value > 0.05, *Z*-score: 2.60) and RDCs, although these sites are extensively researched and well known in the context of genome stability and DSB formation in somatic cells (Figure 3A).

Since DSBs are intentionally induced in multiple stages during spermatogenesis and the efficiency of the subsequent repair is thought to influence the downstream development of the sperm drastically, especially in the context of histone-to-protamine transition and maturation, we further investigated the association of the sperm RDCs in the mature spermatozoa with regions of histone modifications of different stages during spermatogenesis. The publicly available data examined in this study was generated from pachytene spermatocytes, which occur during the first meiotic division when DSBs are induced to enable crossovers, and round spermatids, which occur just before the onset of DSB induction for the histone-to-protamine transition. The results show that the RDCs display larger overlaps with many histone PTMs from the pachytene stage compared to the round spermatid stage and some overlaps are even larger than with the retained histones in the mature spermatozoa. The RDCs with the largest overlap from the pachytene stage are co-localized with H3K4me1 (*p*-value < 0.05, *Z*-score: 14.38) and H3K27ac (*p*-value < 0.05, *Z*-score: 11.19), both of which are also positively associated with them. Interestingly, the RDCs associated with these PTMs are primarily located in introns (47.47% and 41.33%, respectively), especially in intronic SINEs (48.25% and 54.67%, respectively). The strongest association during the round spermatid stage is observed with H3K27me3 (*p*-value < 0.05, *Z*-score: 15.05) and H3K4me1 (*p*-value < 0.05, *Z*-score: 8.95). Notably, the regions associated with H3K27me3 in mature spermatozoa already display this association in 31.67% of cases in the round spermatid stage but only 13.52% in pachytene stage. This suggests that most of the histones in these regions gain their modification late during spermatogenesis. Notably, concerning the association with RDCs, this trend is even more pronounced. Only 26.61% of RDC and H3K27me3 colocalizations in the mature spermatozoa are already present during pachytene, while 62.1% are observed during the round spermatid stage (Figures 3B, C). Since H3K4me1 and H3K27ac are generally associated with active transcription, we examined the top 10% transcribed genes during both stages and their relationship to the RDCs in mature spermatozoa. The data shows a significant correlation between RDCs and the top 10% of transcribed transcripts in both pachytene spermatocytes and round spermatids (*p*-value < 0.05, *Z*-scores: 4.86 and 3.77, respectively), with similar rates of overlap (11.68% and 12.49%, respectively). No larger or significant overlaps were observed for the RDCs with H3K14ac, H3K27ac and H3K9me3 (*p*-value > 0.05, *Z*-scores: −0.49, −0.02 and 0.38, respectively).

### 3.3 RDCs in somatic cells after inhibition of NHEJ

The unique DSB patterns observed in human spermatozoa, which differ from those in somatic cells but resemble those in murine spermatids, are likely linked to the characteristics of the spermatozoa itself (Burden et al., 2023). The dormant state of spermatozoa with regards to metabolism and transcription should result in fewer DSBs, as metabolic stress and transcription are well-accepted causes of DNA damage (Ui et al., 2020). However, mature spermatozoa are also characterized by the absence of active NHEJ, which is likely due to their genomic structure and the absence of certain relevant factors, although the mechanisms are still under scrutiny (Talibova et al., 2022). To investigate the influence of the absent NHEJ activity on the characteristic DSB landscape of the sperm, we inhibited NHEJ activity in a somatic cell model. Therefore, we used AZD7648 to inhibit DNA-PKc in proliferating IMR90 cells. AZD7648 is a highly selective and potent inhibitor for DNA-PKc, the key regulator of NHEJ. By inhibiting the phosphorylation activity of DNA-PKc, NHEJ can be effectively inactivated, without interfering with the activity of other kinases (Fok et al., 2019). Subsequently, we used the above-mentioned methods to detect DSBs and used untreated IMR90 cells as a background to identify a somatic DSB landscape unique in the absence of NHEJ. After depleting the RDCs common to both the untreated and treated IMR90 cells, we identified 6098 RDCs as AZD7648-sensitive clusters (ASCs) since they only occur after inhibition.

**FIGURE 4:**
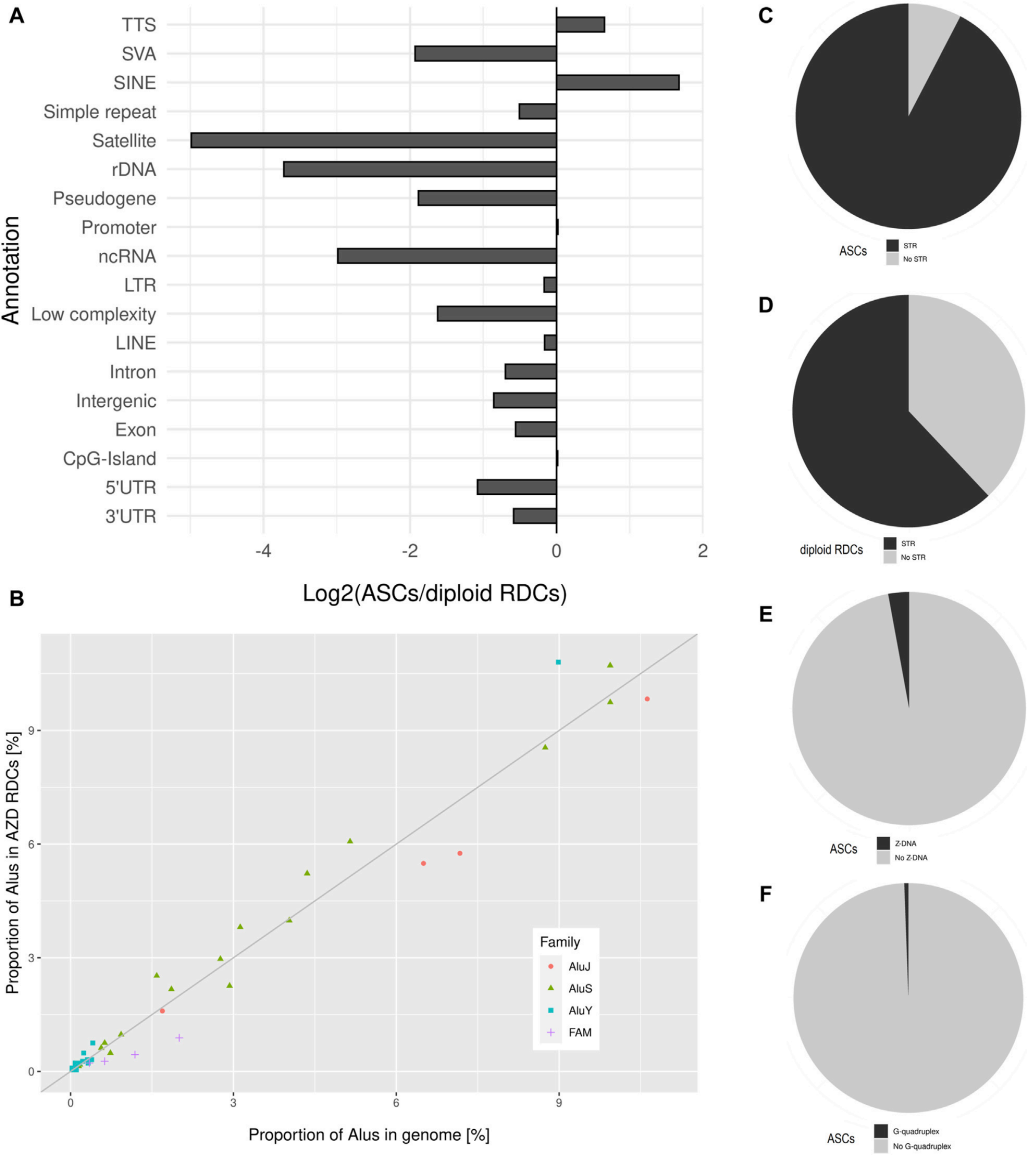
**(A)** Detailed annotation of ASCs as generated by Homer, depicted as log2 (ASC/diploid RDCs) values, which are used to display changes after treatment. **(B)** The distribution of the ASCs across various Alu subfamilies within the human genome is depicted as a proportion of Alu subfamilies located in the ASCs on the *y*-axis, and the relative proportion of the corresponding Alu-subfamily of all genomic Alu elements in the genome on the *x*-axis. The bisection indicates an equal distribution of expected and observed Alu-subfamilies in the ASCs corresponding to their genomic representation. Pie charts displaying the proportion of ASCs overlapping **(C)** STRs, **(D)** in comparison to the diploid RDCs overlapping STRs, ASCs overlapping, **(E)** Z-DNA, and **(F)** G-quadruplexes.

The GC content of the combined ASCs is 40.13%, which is similar to the sperm RDCs and reflects the average GC content of the genome. Regarding the distribution of the ASCs in the genome, an underrepresentation on both sex chromosomes, as well as on chr13, chr18, and chr21 can be observed. On the other hand, the strongest overrepresentation is observed on chr7, chr16, chr17, chr19, and chr22. The resulting distribution of the ASCs in the genome is similar to that in the sperm genome, except for chr7, which displays an enrichment common to diploid IMR cells (Figure 1). As for top-level annotation, the majority of ASCs are located in introns (51.16%) and intergenic regions (43.41%). The detailed annotation with Homer, with corresponding values from untreated IMR90 cells, were used to generate log2-fold changes after NHEJ inhibition. Regarding the detailed annotation, most genetic features show a depletion in the ASCs. The most significantly depleted features are rDNA, satellite DNA, ncRNA, SINE-VNTR-ALUs (SVAs), and pseudogenes. Strikingly, the only enrichment of ASCs is found in SINEs and transcription termination site (TTS) (Figure 4A). SINEs are enriched over 3-fold in the ASCs compared to the diploid cells, leading to a colocalization of 38.90% of ASCs with Alu elements. This pattern is also observed in human spermatozoa. To investigate the relationship between Alus and ASCs, we also analyzed the distribution of ASCs among different Alu subfamilies. We calculated the proportions of subfamilies associated with ASCs and correlated them with their corresponding proportions in the human genome. Similar to the pattern observed in the sperm RDCs, the subfamilies AluY and AluS are predominantly overrepresented in the ASCs. This overall trend is even more pronounced comparing the association of Alus and the ASCs to that of the sperm RDCs. The highest overrepresentation is observed in AluYb8, AluYk4, AluYa8, AluYm1, and AluYc3. On the other hand, the older Alu subfamilies, AlusJ and ancestral Alus, are significantly less represented and exhibit the lowest abundance of ASCs with FLAM_A, AluYk11, FRAM, FLAM_C, and FAM (Figure 4B).

This characteristic is shared between the ASCs and the sperm RDCs, in strong contrast to the untreated IMR90 cells. Further testing was conducted to identify potential shared characteristics between these two cell models regarding the association with short tandem repeats. Notably, 92.37% of the ASCs contain at least one STR, resulting in a strong positive association between STRs and ASCs (*p*-value < 0.05, *Z*-score: 93.24). This characteristic, which is shared with sperm RDCs, is not found in untreated IMR90 cells. Therefore, STRs are observed 1.49 times more frequently in ASCs than in diploid cells and are less strongly associated with the RDCs in diploid IMR90 cells (*p*-value < 0.05, *Z*-score: 18.61) (Figures 4C, D). Comparable to the sperm RDCs, the STRs in the ASCs are favored with (YR)_n_ in 31.81% of ASCs, while the (RY)_n_ found in 29.27%. However, the single most enriched motif in the ASCs is (AT)_n_, as opposed to the (CA)_n_ repeat found in mature spermatozoa. Although certain STRs are associated with the formation of Z-DNA, only 3.12% of ASCs are located in regions of predicted Z-DNA (Figure 4E). This results in a negative association of ASCs with Z-DNA (*p*-value < 0.05, *Z*-score: −3.61). Furthermore, with regards to another common non-B DNA structure, ASCs are also infrequently located in regions predicted to form G-quadruplexes (0.56%), also with a negative association (*p*-value < 0.05, *Z*-score: −2.23) (Figure 4F).

## 4 Discussion

### 4.1 Genomic distribution of RDCs in the absence of NHEJ

The activity of transposable elements in the germline has a significant impact on the stability of the genome and the established mechanisms of mammalian spermatogenesis. As noted by Burden et al. (2023), DSBs in murine spermatids are associated with LINE1 elements. Interestingly, in mature human spermatozoa, we observed a stronger colocalization of recurrent DSBs with Alus than with LINEs, indicating a species-specific linkage between DSB accumulation and TEs in the male germline. The murine genome is heavily influenced by LINEs, many of which are still active. In contrast, the human genome only contains a few dozen active LINEs (Beck et al., 2011; Sookdeo et al., 2013). However, the human genome contains thousands of active Alu elements, with many actively retrotransposing. Notably, AluYa5 and AluYb8, which are among the most active Alus in human populations, show the highest overrepresentation in the RDCs. In contrast, older Alu subfamilies tend to be underrepresented in the RDCs (Konkel et al., 2015). Similar trends were observed in murine spermatids in regard with L1MdA and L1MdT, where both subfamilies contain active copies and display the strongest association with DSBs (Burden et al., 2023). The activity of both TEs involves active transcription and often leads to genomic remodeling, with e.g., many of the mentioned young Alus in humans tend to remain hypomethylated during epigenetic reprogramming (Molaro et al., 2011). This suggests that the activity of these young active Alu elements, and LINEs in the murine genome, may cause the accumulation of DSBs in these elements. The activity of these TEs may result in locally less condensed chromatin compared to the genome-wide highly condensed protamine-packaged chromatin. Furthermore, the ectopic transcription of these TEs may cause DNA damage through transcription-coupled mechanisms, which could lead to errors in the histone-to-protamine transition during spermiogenesis, resulting in regions retaining histones. Furthermore, these “protamine-free regions” could remain transcriptionally active for prolonged periods, even when the rest of the genome is dormant. Taken together, these findings suggest an activity-coupled, species-specific association between DSB accumulation and TEs in the male germline.

On the other hand, we observed an interspecific shared feature of DSB association in human and murine spermatozoa. The in human spermatozoa observed DSBs display the same strong association with short tandem repeats as observed in murine spermatids. Repeats of the motif (YR)_n_, especially (CA)_n_, were strongly favored in both species (Burden et al., 2023). However, an association with Z-DNA, potentially formed by the repetitive nature of STRs, could not be observed in mature human spermatozoa. Although spermatogenesis in humans and mice is very similar, there are some key differences that result in slightly different genomic structures, potentially explaining the differences in association with Z-DNA (Evenson et al., 1980; Yamaguchi et al., 2018).

Remarkably, the strong association between recurrent DSBs and STR can also be induced in a somatic cell model by inhibiting DNA-PKc and thus NHEJ activity. When DNA-PKc was inhibited with AZD7648 in IMR90 cells, the landscape of DSBs differed significantly from that of untreated cells, which is canonically dominated by transcription and accessible chromatin (Mourad et al., 2018). As a result, the DSBs landscape of IMR90 cells in the absence of NHEJ shared characteristics with the RDCs observed in mature spermatozoa. The observed differences in the disposition of DSBs generation and chromatin between spermatozoa and somatic cells suggest that the repetitive nature of STRs and Non-B DNA structures may not be the sole cause of DSBs in human and murine spermatozoa. Furthermore, the absence of NHEJ activity may contribute to these characteristics. The presence of DSBs in both spermatozoa and somatic cells, in the absence of NHEJ activity, suggests that the lack of NHEJ shapes the distinctive DSB landscape in spermatozoa, particularly in STRs and TEs.

### 4.2 Chromatin in mature spermatozoa and TMEJ

Since chromatin status has a large influence on genome stability and also on the functionality of mature spermatozoa we analyzed the chromatin state of the mature spermatozoa in the face of DNA damage (Torres-Flores and Hernández-Hernández, 2020). Interestingly, the colocalization of RDCs and retained histones in mature spermatozoa is limited and the RDCs display stronger positive associations with histone PTMs associated with active transcription during spermatogenesis, such as H3K4me1 and H3K27ac, than with the retained histones in mature spermatozoa. Furthermore, regions associated with R-loops and accessible chromatin do not display an association with recurrent DSB formation, a relationship often observed in somatic cells. This trend is partially carried over to the mature spermatozoa with retained H3K4me1 and H3K36me3 displaying slight positive associations with the RDCs, although mature spermatozoa are generally considered transcriptionally inactive. However, histone PTMs facilitating transcription often lead to less condensed chromatin, which is generally more susceptible to DNA damage, especially when compared to the highly condensed chromatin associated with protamine (Aitken and Iuliis, 2010; Crossley et al., 2019). This could potentially lead to a positive association between less condensed chromatin and DNA damage, rather than active transcription. Strikingly, the strongest association of recurrent DSBs and retained histones in the mature spermatozoa is observed with H3K27me3, which is already observed during spermatogenesis, with H3K27me3 during round spermatid stage displaying the strongest association of all histone PTMs. Since this strong association is not observed during pachytene stage, H3K27me3 seems to arise late during spermatogenesis, particularly during and after the round spermatid stage. This suggests that H3K27me3 may play a role in the generation of DSBs to facilitate the histone-to-protamine transition, since most H3K27me3 is found in round spermatids but not pachytene spermatids.

Furthermore, this trend is even more pronounced in the context of colocalization of recurrent DSBs and H3K27me3. In somatic cells, H3K27me3 is typically found in heterochromatin. However, during spermatogenesis, it also causes the retention of histones, resulting in protamine-free regions near H3K27me3. This leads to less condensed chromatin, although it is still more condensed than, e.g., H3K4me1 or H3K27ac. In addition, H3K27me3 is involved in the early stages of DNA repair, potentially due to its repressive property in the context of transcription and it appears to strongly influence DSB repair in favor of MMEJ over NHEJ (Schep et al., 2021). We propose that during spermatogenesis, Polθ leaves a microsatellite mutational signature associated with erroneous DSB repair by MMEJ, resulting in the addition of short sequences reminiscent of Polθ-associated mechanisms, leading to microsatellite instability, which is often observed in a tumor environment (Wood and Doublié, 2016; Matsuno et al., 2019). Moreover, MMEJ, especially TMEJ, is in fact the most prominent DSB repair mechanism in the male pronucleus of the zygote and the mutational footprint of the involved Polθ can be observed in human populations (Wang et al., 2023).

As some NHEJ factors may be absent after sperm maturation, H3K27me3 could potentially mark genomic regions that undergo recurrent DNA damage during spermatogenesis for reliable repair in the zygote after fertilization. As a result, TMEJ, which is favored by H3K27me3, would repair the recurrent DSBs in STRs and TEs in the male pronucleus of the zygote, rather than using NHEJ in the late stages of spermatogenesis. This would not only ensure an intact genome for the drastic genomic repackaging in the zygote, reversing the histone-to-protamine transition, but also result in high mutational rates due to the mutational rate of Polθ. Thus, regions of recurrent DSB formation through transposable element activity and colocalization of H3K27me3 could result in mutational hotspots. As transcription-coupled damage is mainly found in the 5′-proximal regions of genes, the recurrent occurrence of DSBs in Alus, combined with faulty TMEJ repair, could potentially result in the degradation of the regulatory A- and B-Boxes, which function as RNA polymerase III promoters (Konkel et al., 2015; Wyatt et al., 2016; Dellino et al., 2019). This, in turn, could increase diversity in regulatory elements of species-specific actively transcribed TEs in the male germline over generations on a population scale and as a result lead to the inactivation of young, active TEs in mammalian genomes.

## Data Availability

The original data presented in the study are deposited in SRA, accession number PRJNA1097619, and in GEO, accession number GSE269135. Publicly available datasets were analyzed in this study. The names of the repository/repositories and accession number(s) can be found in the Supplementary Material.
